# Notes and News

**Published:** 1900-02-10

**Authors:** 


### Notes and News.

A new municipal lunatic asylum is to be erected near Berlin for 1,500 patients. The plans which have been submitted for approval will work out at a cost of upwards of £340 per bed.

A new hospital for the employés of the London and North-Western Railway is to be erected at Crewe. It will contain 40 beds, operating-room, isolation ward, and surgeon's quarters.

The staff of the Irish field hospital, presented by Lord Iveagh to the troops in South Africa, will leave on Friday. Sir William Thomson has already started, so as to be prepared with the necessary arrangements when the staff arrives in Cape Town.

Lord Airlie has given a site of 21 acres on his Auchterhouse estate to Dundee for a sanatorium for consumptives. Ex-Provost Moncur, who has given £10,000 for the building, has been appointed a trustee, and plans are to be now prepared.

The Hon. Alec York has placed his house, which adjoins Netley Hospital, at the disposal of the authorities for the use of sick soldiers; and, further, he has thrown open the grounds attached to it for the benefit of the convalescents.

The Queen of Italy will be patroness of the Congress on Tuberculosis to be held at Naples in April. The interest shown by Queen Marguerite is emulated by the Queen of Greece, who is going to found a hospital for consumptives near Athens.

A patient, who had been ill from scarlatina, was recently fined in Ireland for refusing to go into hospital. It is evident that such endangering of public safety is not lightly regarded in Ireland, for the magistrate said that if another similar proceeding came before him he should exact the full penalty of the law.

In spite of the depressing effects of the famine in India, Bombay is the only centre where any increase in the plague is reported. In other quarters there is a distinct decline.

The Council of the Odontological Society of Great Britain has issued a series of regulations in accordance with which grants will be made for the furtherance of scientific research in connection with dentistry.

Endeavours are being made to persuade the militia volunteers and yeomanry to consent to vaccination against typhoid fever. It will be interesting to observe the result of the inoculation which has been so largely resorted to by the troops in the present campaign.

Edinburgh is going to equip a base hospital for the troops in South Africa. The Lord Provost of Edinburgh and General Chapman, commanding the forces in Scotland, are conducting the scheme. One hundred beds will be provided, and the whole of the staff will be drawn from Edinburgh.

We record with great regret the death of Dr. Francis Charlwood Turner, physician to the London Hospital. He was the son of Mr. Thomas Turner, who for twenty years was treasurer to Guy's Hospital, and it was at Guy's and at Cambridge that he received his medical education. Dr. Charlwood Turner was a well-known contributor to medical literature, and will be greatly missed.

An International Congress of Medical Electrology and Radiology will take place in Paris from July 27th to August 1st, 1900. A commission, which is composed of Messrs. Weiss, Professor at the University of Paris (president), Apostoli and Oudin (vice-presidents), Doumer, Professor at the University of Lille (general secretary), Moutier (secretary), Boisseau du Rocher (treasurer); and of Messrs. Bergonié (Professor at the University of Bordeaux), Bouchacourt, Branly (Professor at the Catholic Institute of Paris), Larat, Radiguet, Villemin (surgeon of the hospitals of Paris), has been asked to undertake its organisation. All inquiries for further information must be forwarded to Professor E. Doumer, general secretary, 57, Rue Nicolas-Leblanc, Lille.

By the death of Sir Thomas Grainger-Stewart, Professor of the Practice of Physic and of Clinical Medicine at the University of Edinburgh, Scotland loses one of its most distinguished medical men. He received his medical education at Edinburgh, Berlin, Prague, and Vienna. He occupied various posts at the Edinburgh Royal Infirmary, finally becoming Professor of the Practice of Physic, which post he occupied until his death. He was President of the Royal College of Physicians, Edinburgh, from 1889 to 1891, he received the honorary degree of LL.D. at Aberdeen, was physician in ordinary to the Queen, and was created a knight in 1894. He wrote a great deal on special medical topics, and his work on Bright's disease has become widely known. Professor Grainger-Stewart died at the age of 63 years.

